# Editorial for SARS-CoV-2 and COVID-19 Topical Collection

**DOI:** 10.3390/v16030356

**Published:** 2024-02-25

**Authors:** Luis Martinez-Sobrido, Fernando Almazán

**Affiliations:** 1 Texas Biomedical Research Institute, San Antonio, TX 78227, USA; 2 Department of Molecular and Cellular Biology, Centro Nacional de Biotecnología (CNB), CSIC, 28049 Madrid, Spain

A previously unknown coronavirus, severe acute respiratory syndrome coronavirus 2 (SARS-CoV-2), was isolated in Wuhan, China in December 2019, from a patient with a respiratory disease linked to potential contact with wild animals. The emergence of SARS-CoV-2 infection in humans resulted in the coronavirus disease 2019 (COVID-19) pandemic, which has had an alarming case fatality rate and has posed a threat to human health and socioeconomic activity across the world. The multinational efforts to develop vaccines have resulted in several candidates with excellent safety and efficacy profiles, and large-scale vaccination campaigns were implemented early in 2021 to prevent SARS-CoV-2 infections. Likewise, several antivirals have been developed and are currently used in the therapeutic treatment of the SARS-CoV-2 infection.

Since the emergence of SARS-CoV-2, unprecedented massive advances have been made in practically all research areas regarding the biology of SARS-CoV-2 and its associated COVID-19 disease. In this Topical Collection, “SARS-CoV-2 and COVID-19”, we aimed to cover different aspects related to molecular biology, viral replication and transmission, virus–host interactions, viral pathogenesis, virus epidemiology, virus evolution, prophylactic vaccines, therapeutic antivirals, viral detection and diagnostics, and clinical studies in SARS-CoV-2 infection and COVID-19 disease, with the overall goal of providing the latest insights into this important human pathogen and its associated disease. For this Topical Collection, we have assembled a total of 101 manuscripts, including 4 review articles, 5 brief reports, 9 communications, 3 case reports, 1 opinion, 1 perspective, and 78 original research articles.

The first part of this Topical Collection is focused on the molecular biology of SARS-CoV-2. This section is covered by two original manuscripts exploring conformational landscapes and cryptic binding pockets in distinct functional states of the SARS-CoV-2 Omicron BA.1 and BA.2 variants of concern (VoCs) (1) and a second manuscript describing a computational model of the SARS-CoV-2 Spike (S) protein and its implications for the viral membrane fusion mechanism (2).

The second Topic focuses on the recent advances in viral replication and transmission and comprises one case report addressing multiorgan and vascular tropism in SARS-CoV-2 (3) and four original research articles evaluating the different outcomes of the original virus and the B.1.617.2 and B.1.1529 VoCs of SARS-CoV-2 in transgenic K18 hACE mice (4), the spread of COVID-19 at the United States Department of Veterans Affairs during the beginning of the 2019-2020 influenza season (5), the capacity of arthropod ectoparasites to bind SARS-CoV-2 via ACE (6), and the development of a human A549 cell line expressing ACE2 and TMPRSS2 to facilitate the viral infection of SARS-CoV-2, including VoCs (7).

The third Topic in this Topical Collection relates to virus–host interactions and includes one review article describing ACE2-independent alternative receptors for SARS-CoV-2 (8) and three original research manuscripts describing the expression of SARS-CoV-2 entry factors in patients with chronic hepatitis (9), complement activation-independent attenuation of SARS-CoV-2 infection by C1q and C4b-binding proteins (10), and the balance of the host cell membrane, receptor, and antibody docking in the SARS-CoV-2 Omicron VoC (11).

The fourth subject area in the Topical Collection addresses SARS-CoV-2 pathogenesis. This section is covered by one review article describing hyperinflammatory responses in COVID-19 (12), a brief report on the association between SARS-CoV-2 viral load and patient symptoms and clinical outcomes using droplet digital PCR (13), and twelve original research articles covering different aspects of the pathogenesis of SARS-CoV-2. These articles include studies on the effect of SARS-CoV-2 infection on vagal activity (14), Epstein–Barr virus and human herpesvirus-6 reactivation in COVID-19 patients (15), the avidity of the receptor-binding domain (RBG) IgG as a prognostic factor (16), the prevalence of acute respiratory distress syndrome (ARDS) in hospitalized patients (17), clinical aspects in asymptomatic, moderate, and severe COVID-19 patients (18,19), COVID-19 infection in pregnancy (20), multiorgan virus dissemination (21,22), the antibody-dependent enhancement of SARS-CoV-2 infection (23), the relationship between cardiovascular-disease-associated immune dysregulation and SARS-CoV-2 infection (24), and an analysis of SARS-CoV-2 infection in domestic and wild animals (25).

The fifth section covers the epidemiology of SARS-CoV-2 and includes four communication articles describing viral shedding among re-positive SARS-CoV-2 individuals in the Republic of Korea (26), the lack of evidence for SARS-CoV-2 spillover in free-living neotropical non-human primates in Brazil (27), the neutralizing activity of serum against the B.1.17, B.1.351, and P.1 SARS-CoV-2 VoCs in hospitalized COVID-19 patients (28), and SARS-CoV-2 re-infection in a healthcare worker despite the presence of neutralizing antibodies (29). This section also includes two case reports describing the first detection of the SARS-CoV-2 B.1.1.7 VoC in an asymptomatic dog in Spain (30) and a genomic surveillance of SARS-CoV-2 in Lebanon (31), and eleven original research articles describing different aspects of the epidemiology of SARS-CoV-2 (32–42).

The next section of the Topical Collection focuses on SARS-CoV-2 evolution and contains two review articles describing the evolution of the SARS-CoV-2 Omicron VoC and the genetic impact on viral fitness (43) and the Spike protein of the new SARS-CoV-2 VoCs (44). This section also includes two brief reports on the genomic and temporal analysis of deletions in the nucleocapsid (N) gene of the SARS-CoV-2 Omicron VoC and their correlation with qRT-PCR dropout (45), and the evolution and immune escape mutations in the S gene present in mild or moderate COVID-19 patients treated with monoclonal antibodies (46). Finally, a total of twelve original research articles are included in this part of the Topical Collection, describing different aspects of the evolution of SARS-CoV-2 (47–58).

The seventh Topic in this Topical Collection is mainly focused on vaccines and vaccination studies. This section includes two communication manuscripts describing the decline in the binding capability of the SARS-CoV-2 Omicron VoC B.1.1.529 RBD to antibodies present in convalescent sera and inactivated vaccine sera early in the COVID-19 pandemic (59) and the T cell responses elicited by COVID-19 vaccines or infection against the SARS-CoV-2 Omicron VoC (60), an opinion letter describing how repeated exposure to sub-infectious doses of SARS-CoV-2 promotes T cell immunity and protection against severe COVID-19 (61), and a perspective on the roles of adjuvants in COVID-19 vaccines (62). This section also contains seven original research manuscripts covering analyses of different adjuvants in SARS-CoV-2 vaccines (63) and different aspects of COVID-19 vaccination (64–69).

The next section focuses on SARS-CoV-2 antivirals and treatments and includes two communications describing the antiviral activity of liposomal lactoferrin against HCoV-229E and SARS-CoV-2 pseudotyped viruses in vitro (70) and the safety and effectiveness of Molnupiravir against SARS-CoV-2 in hemodialyzed patients and kidney transplant recipients (71), and ten original research documents describing different aspects of antiviral research against SARS-CoV-2 in vitro or in validated animal models of viral infection (72–81).

The next section includes one brief report on performance of self-collected saliva testing compared with nasopharyngeal swab testing for the detection of SARS-CoV-2 (82), one communication describing a new high-throughput nanopore-sequencing strategy for the rapid and automated typing of the SARS-CoV-2 S protein (83), and thirteen original research articles covering different aspects of SARS-CoV-2 detection and/or virus diagnosis (84–96).

The last section of this Topical Collection covers different clinical studies related to SARS-CoV-2 performed in different countries and scenarios. These include a brief report on the burden of pediatric SARS-CoV-2 hospitalizations during the Omicron wave in Germany (97), and four research articles describing the risk factors for the hospitalization of COVID-19 patients in the GENCOV study (98), an analysis of the variability in the clinical course of COVID-19 using a large real-world database (99), a characterization of hematological changes in COVID-19 patients (100), and an analysis of incubation and acute disease stages in mild-to-moderate COVID-19 patients (101).

We hope that the different articles published in this Topical Collection offers readers a comprehensive view of the most recent advances in SARS-CoV-2 and COVID-19 research and stimulates future research, as well as open fruitful collaborations, to increase our current understanding of this important human respiratory pathogen, with the goal of developing more efficient prophylactics and/or therapeutics for the efficient control of SARS-CoV-2 infection and associated COVID-19 disease. We would like to thank all the contributing authors for their time, effort, and participation in this Topical Collection. We would also like to thank the Editorial Office at *Viruses* for all their help, support, and advice in putting together this Topical Collection.

## List of Contributions

1. Verkhivker, G.; Alshahrani, M.; Gupta, G. Exploring Conformational Landscapes and Cryptic Binding Pockets in Distinct Functional States of the SARS-CoV-2 Omicron BA.1 and BA.2 Trimers: Mutation-Induced Modulation of Protein Dynamics and Network-Guided Prediction of Variant-Specific Allosteric Binding Sites. *Viruses* **2023**, *15*, 9. https://doi.org/10.3390/v15102009.
2. Nishima, W.; Kulik, M. Full-Length Computational Model of the SARS-CoV-2 Spike Protein and Its Implications for a Viral Membrane Fusion Mechanism. *Viruses* **2021**, *13*, 1126. https://doi.org/10.3390/v13061126.
3. Hartard, C.; Chaqroun, A.; Settembre, N.; Gauchotte, G.; Lefevre, B.; Marchand, E.; Mazeaud, C.; Nguyen, D.T.; Martrille, L.; Koscinski, I.; et al. Multiorgan and Vascular Tropism of SARS-CoV-2. *Viruses* **2022**, *14*, 515. https://doi.org/10.3390/v14030515.
4. He, Y.; Henley, J.; Sell, P.; Comai, L. Differential Outcomes of Infection by Wild-Type SARS-CoV-2 and the B.1.617.2 and B.1.1.529 Variants of Concern in K18-hACE2 Transgenic Mice. *Viruses* **2023**, *16*, 60. https://doi.org/10.3390/v16010060.
5. Lukowsky, L.R.; Der-Martirosian, C.; Steers, W.N.; Kamble, K.S.; Dobalian, A. Using an Administrative and Clinical Database to Determine the Early Spread of COVID-19 at the US Department of Veterans Affairs during the Beginning of the 2019–2020 Flu Season: A Retrospective Longitudinal Study. *Viruses* **2022**, *14*, 200. https://doi.org/10.3390/v14020200.
6. Lam, S.D.; Ashford, P.; Diaz-Sanchez, S.; Villar, M.; Gortazar, C.; de la Fuente, J.; Orengo, C. Arthropod Ectoparasites Have Potential to Bind SARS-CoV-2 via ACE. *Viruses* **2021**, *13*, 708. https://doi.org/10.3390/v13040708.
7. Chang, C.W.; Parsi, K.M.; Somasundaran, M.; Vanderleeden, E.; Liu, P.; Cruz, J.; Cousineau, A.; Finberg, R.W.; Kurt-Jones, E.A. A Newly Engineered A549 Cell Line Expressing ACE2 and TMPRSS2 Is Highly Permissive to SARS-CoV-2, Including the Delta and Omicron Variants. *Viruses* **2022**, *14*, 1369. https://doi.org/10.3390/v14071369.
8. Lim, S.; Zhang, M.; Chang, T.L. ACE2-Independent Alternative Receptors for SARS-CoV-2. *Viruses* **2022**, *14*, 2535. https://doi.org/10.3390/v14112535.
9. Rosso, C.; Demelas, C.; Agostini, G.; Abate, M.L.; Vernero, M.; Caviglia, G.P.; D’Amato, D.; Armandi, A.; Tapparo, M.; Guariglia, M.; et al. Expression of SARS-CoV-2 Entry Factors in Patients with Chronic Hepatitis. *Viruses* **2022**, *14*, 2397. https://doi.org/10.3390/v14112397.
10. Beirag, N.; Varghese, P.M.; Neto, M.M.; Al Aiyan, A.; Khan, H.A.; Qablan, M.; Shamji, M.H.; Sim, R.B.; Temperton, N.; Kishore, U. Complement Activation-Independent Attenuation of SARS-CoV-2 Infection by C1q and C4b-Binding Protein. *Viruses* **2023**, *15*, 1269. https://doi.org/10.3390/v15061269.
11. Overduin, M.; Bhat, R.K.; Kervin, T.A. SARS-CoV-2 Omicron Subvariants Balance Host Cell Membrane, Receptor, and Antibody Docking via an Overlapping Target Site. *Viruses* **2023**, *15*, 447. https://doi.org/10.3390/v15020447.
12. Silva, M.J.A.; Ribeiro, L.R.; Gouveia, M.I.M.; Marcelino, B.D.R.; Santos, C.S.D.; Lima, K.V.B.; Lima, L. Hyperinflammatory Response in COVID-19: A Systematic Review. *Viruses* **2023**, *15*, 553. https://doi.org/10.3390/v15020553.
13. Hastie, E.; Amogan, H.; Looney, D.; Mehta, S.R. Association between SARS-CoV-2 Viral Load and Patient Symptoms and Clinical Outcomes Using Droplet Digital PCR. *Viruses* **2023**, *15*, 446. https://doi.org/10.3390/v15020446.
14. Acanfora, D.; Nolano, M.; Acanfora, C.; Colella, C.; Provitera, V.; Caporaso, G.; Rodolico, G.R.; Bortone, A.S.; Galasso, G.; Casucci, G. Impaired Vagal Activity in Long-COVID-19 Patients. *Viruses* **2022**, *14*, 1035. https://doi.org/10.3390/v14051035.
15. Brooks, B.; Tancredi, C.; Song, Y.; Mogus, A.T.; Huang, M.W.; Zhu, H.; Phan, T.L.; Zhu, H.; Kadl, A.; Woodfolk, J.; et al. Epstein-Barr Virus and Human Herpesvirus-6 Reactivation in Acute COVID-19 Patients. *Viruses* **2022**, *14*, 1872. https://doi.org/10.3390/v14091872.
16. Manuylov, V.; Burgasova, O.; Borisova, O.; Smetanina, S.; Vasina, D.; Grigoriev, I.; Kudryashova, A.; Semashko, M.; Cherepovich, B.; Kharchenko, O.; et al. Avidity of IgG to SARS-CoV-2 RBD as a Prognostic Factor for the Severity of COVID-19 Reinfection. *Viruses* **2022**, *14*, 617. https://doi.org/10.3390/v14030617.
17. Gujski, M.; Jankowski, M.; Rabczenko, D.; Gorynski, P.; Juszczyk, G. The Prevalence of Acute Respiratory Distress Syndrome (ARDS) and Outcomes in Hospitalized Patients with COVID-19-A Study Based on Data from the Polish National Hospital Register. *Viruses* **2022**, *14*, 76. https://doi.org/10.3390/v14010076.
18. Bae, S.; Kim, J.Y.; Lim, S.Y.; Park, H.; Cha, H.H.; Kwon, J.S.; Suh, M.H.; Lee, H.J.; Lim, J.S.; Jung, J.; et al. Dynamics of Viral Shedding and Symptoms in Patients with Asymptomatic or Mild COVID-19. *Viruses* **2021**, *13*, 2133. https://doi.org/10.3390/v13112133.
19. Golovkin, A.; Kalinina, O.; Bezrukikh, V.; Aquino, A.; Zaikova, E.; Karonova, T.; Melnik, O.; Vasilieva, E.; Kudryavtsev, I. Imbalanced Immune Response of T-Cell and B-Cell Subsets in Patients with Moderate and Severe COVID-19. *Viruses* **2021**, *13*, 1966. https://doi.org/10.3390/v13101966.
20. Laresgoiti-Servitje, E.; Cardona-Perez, J.A.; Hernandez-Cruz, R.G.; Helguera-Repetto, A.C.; Valdespino-Vazquez, M.Y.; Moreno-Verduzco, E.R.; Villegas-Mota, I.; Acevedo-Gallegos, S.; Rodriguez-Bosch, M.; Leon-Juarez, M.; et al. COVID-19 Infection in Pregnancy: PCR Cycle Thresholds, Placental Pathology, and Perinatal Outcomes. *Viruses* **2021**, *13*, 1884. https://doi.org/10.3390/v13091884.
21. Odilov, A.; Volkov, A.; Abdullaev, A.; Gasanova, T.; Lipina, T.; Babichenko, I. COVID-19: Multiorgan Dissemination of SARS-CoV-2 Is Driven by Pulmonary Factors. *Viruses* **2021**, *14*, 39. https://doi.org/10.3390/v14010039.
22. Abdullaev, A.; Odilov, A.; Ershler, M.; Volkov, A.; Lipina, T.; Gasanova, T.; Lebedin, Y.; Babichenko, I.; Sudarikov, A. Viral Load and Patterns of SARS-CoV-2 Dissemination to the Lungs, Mediastinal Lymph Nodes, and Spleen of Patients with COVID-19 Associated Lymphopenia. *Viruses* **2021**, *13*, 1410. https://doi.org/10.3390/v13071410.
23. Shen, X.R.; Li, Q.; Li, H.L.; Wang, X.; Wang, Q.; Zheng, X.S.; Geng, R.; Zhang, Y.L.; Li, B.; Jiang, R.D.; et al. Antibody-Dependent Enhancement of SARS-CoV-2 Infection of Human Immune Cells: In Vitro Assessment Provides Insight in COVID-19 Pathogenesis. *Viruses* **2021**, *13*, 2483. https://doi.org/10.3390/v13122483.
24. Lee, A.C.; Castaneda, G.; Li, W.T.; Chen, C.; Shende, N.; Chakladar, J.; Taub, P.R.; Chang, E.Y.; Ongkeko, W.M. COVID-19 Severity Potentially Modulated by Cardiovascular-Disease-Associated Immune Dysregulation. *Viruses* **2021**, *13*, 1018. https://doi.org/10.3390/v13061018.
25. Farber, I.; Kruger, J.; Rocha, C.; Armando, F.; von Kockritz-Blickwede, M.; Pohlmann, S.; Braun, A.; Baumgartner, W.; Runft, S.; Kruger, N. Investigations on SARS-CoV-2 Susceptibility of Domestic and Wild Animals Using Primary Cell Culture Models Derived from the Upper and Lower Respiratory Tract. *Viruses* **2022**, *14*, 828. https://doi.org/10.3390/v14040828.
26. Kim, J.M.; Ryu, B.; Choe, Y.J.; Jo, H.J.; Lee, H.; Kim, H.M.; Lee, N.J.; Rhee, J.E.; Chung, Y.S.; Han, M.G.; et al. Viral Shedding among Re-Positive Severe Acute Respiratory Syndrome Coronavirus-2 Positive Individuals in Republic of Korea. *Viruses* **2021**, *13*, 2089. https://doi.org/10.3390/v13102089.
27. Sacchetto, L.; Chaves, B.A.; Costa, E.R.; de Menezes Medeiros, A.S.; Gordo, M.; Araujo, D.B.; Oliveira, D.B.L.; da Silva, A.P.B.; Negri, A.F.; Durigon, E.L.; et al. Lack of Evidence of Severe Acute Respiratory Syndrome Coronavirus 2 (SARS-CoV-2) Spillover in Free-Living Neotropical Non-Human Primates, Brazil. *Viruses* **2021**, *13*, 1933. https://doi.org/10.3390/v13101933.
28. Trombetta, C.M.; Marchi, S.; Viviani, S.; Manenti, A.; Benincasa, L.; Ruello, A.; Bombardieri, E.; Vicenti, I.; Zazzi, M.; Montomoli, E. Serum Neutralizing Activity against B.1.1.7, B.1.351, and P.1 SARS-CoV-2 Variants of Concern in Hospitalized COVID-19 Patients. *Viruses* **2021**, *13*, 1347. https://doi.org/10.3390/v13071347.
29. Brehm, T.T.; Pfefferle, S.; von Possel, R.; Kobbe, R.; Norz, D.; Schmiedel, S.; Grundhoff, A.; Olearo, F.; Emmerich, P.; Robitaille, A.; et al. SARS-CoV-2 Reinfection in a Healthcare Worker Despite the Presence of Detectable Neutralizing Antibodies. *Viruses* **2021**, *13*, 661. https://doi.org/10.3390/v13040661.
30. Barroso-Arevalo, S.; Rivera, B.; Dominguez, L.; Sanchez-Vizcaino, J.M. First Detection of SARS-CoV-2 B.1.1.7 Variant of Concern in an Asymptomatic Dog in Spain. *Viruses* **2021**, *13*, 1379. https://doi.org/10.3390/v13071379.
31. Nour, D.; Rafei, R.; Lamarca, A.P.; de Almeida, L.G.P.; Osman, M.; Ismail, M.B.; Mallat, H.; Berry, A.; Burfin, G.; Semanas, Q.; et al. The Role of Lebanon in the COVID-19 Butterfly Effect: The B.1.398 Example. *Viruses* **2022**, *14*, 1640. https://doi.org/10.3390/v14081640.
32. Hulst, M.; Kant, A.; Harders-Westerveen, J.; Hoffmann, M.; Xie, Y.; Laheij, C.; Murk, J.L.; Van der Poel, W.H.M. Cross-Reactivity of Human, Wild Boar, and Farm Animal Sera from Pre- and Post-Pandemic Periods with Alpha- and Betaeta-Coronaviruses (CoV), including SARS-CoV-2. *Viruses* **2023**, *16*, 34. https://doi.org/10.3390/v16010034.
33. Einhauser, S.; Peterhoff, D.; Beileke, S.; Gunther, F.; Niller, H.H.; Steininger, P.; Knoll, A.; Korn, K.; Berr, M.; Schutz, A.; et al. Time Trend in SARS-CoV-2 Seropositivity, Surveillance Detection- and Infection Fatality Ratio until Spring 2021 in the Tirschenreuth County-Results from a Population-Based Longitudinal Study in Germany. *Viruses* **2022**, *14*, 1168. https://doi.org/10.3390/v14061168.
34. Wagner, R.; Peterhoff, D.; Beileke, S.; Gunther, F.; Berr, M.; Einhauser, S.; Schutz, A.; Niller, H.H.; Steininger, P.; Knoll, A.; et al. Estimates and Determinants of SARS-CoV-2 Seroprevalence and Infection Fatality Ratio Using Latent Class Analysis: The Population-Based Tirschenreuth Study in the Hardest-Hit German County in Spring 2020. *Viruses* **2021**, *13*, 1118. https://doi.org/10.3390/v13061118.
35. Scozzari, G.; Costa, C.; Migliore, E.; Coggiola, M.; Ciccone, G.; Savio, L.; Scarmozzino, A.; Pira, E.; Cassoni, P.; Galassi, C.; et al. Prevalence, Persistence, and Factors Associated with SARS-CoV-2 IgG Seropositivity in a Large Cohort of Healthcare Workers in a Tertiary Care University Hospital in Northern Italy. *Viruses* **2021**, *13*, 1064. https://doi.org/10.3390/v13061064.
36. Dings, C.; Gotz, K.M.; Och, K.; Sihinevich, I.; Werthner, Q.; Smola, S.; Bliem, M.; Mahfoud, F.; Volk, T.; Kreuer, S.; et al. Model-Based Analysis of SARS-CoV-2 Infections, Hospitalization and Outcome in Germany, the Federal States and Districts. *Viruses* **2022**, *14*, 2114. https://doi.org/10.3390/v14102114.
37. Palyanova, N.; Sobolev, I.; Alekseev, A.; Glushenko, A.; Kazachkova, E.; Markhaev, A.; Kononova, Y.; Gulyaeva, M.; Adamenko, L.; Kurskaya, O.; et al. Genomic and Epidemiological Features of COVID-19 in the Novosibirsk Region during the Beginning of the Pandemic. *Viruses* **2022**, *14*, 2036. https://doi.org/10.3390/v14092036.
38. Silva, J.P.; de Lima, A.B.; Alvim, L.B.; Malta, F.S.V.; Mendonca, C.; Fonseca, P.L.C.; Moreira, F.R.R.; Queiroz, D.C.; Ferreira, J.G.G.; Ferreira, A.C.S.; et al. Delta Variant of SARS-CoV-2 Replacement in Brazil: A National Epidemiologic Surveillance Program. *Viruses* **2022**, *14*, 847. https://doi.org/10.3390/v14050847.
39. Martinez, M.; Nguyen, P.V.; Su, M.; Cardozo, F.; Valenzuela, A.; Franco, L.; Galeano, M.E.; Rojas, L.E.; Diaz Acosta, C.C.; Fernandez, J.; et al. SARS-CoV-2 Variants in Paraguay: Detection and Surveillance with an Economical and Scalable Molecular Protocol. *Viruses* **2022**, *14*, 873. https://doi.org/10.3390/v14050873.
40. Cahyani, I.; Putro, E.W.; Ridwanuloh, A.M.; Wibowo, S.; Hariyatun, H.; Syahputra, G.; Akbariani, G.; Utomo, A.R.; Ilyas, M.; Loose, M.; et al. Genome Profiling of SARS-CoV-2 in Indonesia, ASEAN and the Neighbouring East Asian Countries: Features, Challenges and Achievements. *Viruses* **2022**, *14*, 778. https://doi.org/10.3390/v14040778.
41. Goryoka, G.W.; Cossaboom, C.M.; Gharpure, R.; Dawson, P.; Tansey, C.; Rossow, J.; Mrotz, V.; Rooney, J.; Torchetti, M.; Loiacono, C.M.; et al. One Health Investigation of SARS-CoV-2 Infection and Seropositivity among Pets in Households with Confirmed Human COVID-19 Cases-Utah and Wisconsin, 2020. *Viruses* **2021**, *13*, 1813. https://doi.org/10.3390/v13091813.
42. Stevanovic, V.; Tabain, I.; Vilibic-Cavlek, T.; Mauric Maljkovic, M.; Benvin, I.; Hruskar, Z.; Kovac, S.; Smit, I.; Miletic, G.; Hadina, S.; et al. The Emergence of SARS-CoV-2 within the Dog Population in Croatia: Host Factors and Clinical Outcome. *Viruses* **2021**, *13*, 1430. https://doi.org/10.3390/v13081430.
43. Liu, W.; Huang, Z.; Xiao, J.; Wu, Y.; Xia, N.; Yuan, Q. Evolution of the SARS-CoV-2 Omicron Variants: Genetic Impact on Viral Fitness. *Viruses* **2024**, *16*, 184.
44. Winger, A.; Caspari, T. The Spike of Concern-The Novel Variants of SARS-CoV-2. *Viruses* **2021**, *13*, 1002. https://doi.org/10.3390/v13061002.
45. Gatti, G.; Brandolini, M.; Mancini, A.; Taddei, F.; Zannoli, S.; Dirani, G.; Manera, M.; Arfilli, V.; Denicolo, A.; Marzucco, A.; et al. Genomic and Temporal Analysis of Deletions Correlated to qRT-PCR Dropout in N Gene in Alpha, Delta and Omicron Variants. *Viruses* **2023**, *15*, 1630. https://doi.org/10.3390/v15081630.
46. Jary, A.; Marot, S.; Faycal, A.; Leon, S.; Sayon, S.; Zafilaza, K.; Ghidaoui, E.; Quoc, S.N.; Nemlaghi, S.; Choquet, S.; et al. Spike Gene Evolution and Immune Escape Mutations in Patients with Mild or Moderate Forms of COVID-19 and Treated with Monoclonal Antibodies Therapies. *Viruses* **2022**, *14*, 226. https://doi.org/10.3390/v14020226.
47. Dong, X.; Hiscox, J.A. Analysis of SARS-CoV-2 Population Genetics from Samples Associated with Huanan Market and Early Cases Identifies Substitutions Associated with Future Variants of Concern. *Viruses* **2023**, *15*, 1728. https://doi.org/10.3390/v15081728.
48. Verkhivker, G.; Alshahrani, M.; Gupta, G. Balancing Functional Tradeoffs between Protein Stability and ACE2 Binding in the SARS-CoV-2 Omicron BA.2, BA.2.75 and XBB Lineages: Dynamics-Based Network Models Reveal Epistatic Effects Modulating Compensatory Dynamic and Energetic Changes. *Viruses* **2023**, *15*, 1143. https://doi.org/10.3390/v15051143.
49. Cai, X.; Lan, T.; Ping, P.; Oliver, B.; Li, J. Intra-Host Co-Existing Strains of SARS-CoV-2 Reference Genome Uncovered by Exhaustive Computational Search. *Viruses* **2023**, *15*, 1065. https://doi.org/10.3390/v15051065.
50. Piantham, C.; Ito, K. Predicting the Trajectory of Replacements of SARS-CoV-2 Variants Using Relative Reproduction Numbers. *Viruses* **2022**, *14*, 2556. https://doi.org/10.3390/v14112556.
51. Soares, R.; Vieira, C.P.; Vieira, J. Predictive Models of within- and between-Species SARS-CoV-2 Transmissibility. *Viruses* **2022**, *14*, 1565. https://doi.org/10.3390/v14071565.
52. Bamford, C.G.G.; Broadbent, L.; Aranday-Cortes, E.; McCabe, M.; McKenna, J.; Courtney, D.G.; Touzelet, O.; Ali, A.; Roberts, G.; Lopez Campos, G.; et al. Comparison of SARS-CoV-2 Evolution in Paediatric Primary Airway Epithelial Cell Cultures Compared with Vero-Derived Cell Lines. *Viruses* **2022**, *14*, 325. https://doi.org/10.3390/v14020325.
53. Foster, C.S.P.; Stelzer-Braid, S.; Deveson, I.W.; Bull, R.A.; Yeang, M.; Au, J.P.; Ruiz Silva, M.; van Hal, S.J.; Rockett, R.J.; Sintchenko, V.; et al. Assessment of Inter-Laboratory Differences in SARS-CoV-2 Consensus Genome Assemblies between Public Health Laboratories in Australia. *Viruses* **2022**, *14*, 185. https://doi.org/10.3390/v14020185.
54. Pucci, F.; Rooman, M. Prediction and Evolution of the Molecular Fitness of SARS-CoV-2 Variants: Introducing SpikePro. *Viruses* **2021**, *13*, 935. https://doi.org/10.3390/v13050935.
55. Jaspe, R.C.; Loureiro, C.L.; Sulbaran, Y.; Moros, Z.C.; D’Angelo, P.; Hidalgo, M.; Rodriguez, L.; Alarcon, V.; Aguilar, M.; Sanchez, D.; et al. Description of a One-Year Succession of Variants of Interest and Concern of SARS-CoV-2 in Venezuela. *Viruses* **2022**, *14*, 1378. https://doi.org/10.3390/v14071378.
56. Capozzi, L.; Bianco, A.; Del Sambro, L.; Simone, D.; Lippolis, A.; Notarnicola, M.; Pesole, G.; Pace, L.; Galante, D.; Parisi, A. Genomic Surveillance of Circulating SARS-CoV-2 in South East Italy: A One-Year Retrospective Genetic Study. *Viruses* **2021**, *13*, 731. https://doi.org/10.3390/v13050731.
57. Xia, X. Dating the Common Ancestor from an NCBI Tree of 83688 High-Quality and Full-Length SARS-CoV-2 Genomes. *Viruses* **2021**, *13*, 1790. https://doi.org/10.3390/v13091790.
58. Zhang, J.; Fan, L.; Xu, H.; Fu, Y.; Peng, X.; Zheng, Y.; Yu, J.; He, J. Evolutionary Pattern Comparisons of the SARS-CoV-2 Delta Variant in Countries/Regions with High and Low Vaccine Coverage. *Viruses* **2022**, *14*, 2296. https://doi.org/10.3390/v14102296.
59. Zhou, W.; He, P.; Li, J.; Liu, H.; Shi, M.; Yu, J.; Wei, H. Steep Decline in Binding Capability of SARS-CoV-2 Omicron Variant (B.1.1.529) RBD to the Antibodies in Early COVID-19 Convalescent Sera and Inactivated Vaccine Sera. *Viruses* **2022**, *14*, 335. https://doi.org/10.3390/v14020335.
60. Ahmed, S.F.; Quadeer, A.A.; McKay, M.R. SARS-CoV-2 T Cell Responses Elicited by COVID-19 Vaccines or Infection Are Expected to Remain Robust against Omicron. *Viruses* **2022**, *14*, 79. https://doi.org/10.3390/v14010079.
61. De Angelis, M.L.; Francescangeli, F.; Rossi, R.; Giuliani, A.; De Maria, R.; Zeuner, A. Repeated Exposure to Subinfectious Doses of SARS-CoV-2 May Promote T Cell Immunity and Protection against Severe COVID-19. *Viruses* **2021**, *13*, 961. https://doi.org/10.3390/v13060961.
62. Zhang, N.; Li, K.; Liu, Z.; Nandakumar, K.S.; Jiang, S. A Perspective on the Roles of Adjuvants in Developing Highly Potent COVID-19 Vaccines. *Viruses* **2022**, *14*, 387. https://doi.org/10.3390/v14020387.
63. Zhang, N.; Ji, Q.; Liu, Z.; Tang, K.; Xie, Y.; Li, K.; Zhou, J.; Li, S.; Shang, H.; Shi, Z.; et al. Effect of Different Adjuvants on Immune Responses Elicited by Protein-Based Subunit Vaccines against SARS-CoV-2 and Its Delta Variant. *Viruses* **2022**, *14*, 501. https://doi.org/10.3390/v14030501.
64. Messchendorp, A.L.; Sanders, J.F.; Abrahams, A.C.; Bemelman, F.J.; Bouwmans, P.; van den Dorpel, R.M.A.; Hilbrands, L.B.; Imhof, C.; Reinders, M.E.J.; Rispens, T.; et al. Incidence and Severity of COVID-19 in Relation to Anti-Receptor-Binding Domain IgG Antibody Level after COVID-19 Vaccination in Kidney Transplant Recipients. *Viruses* **2024**, *16*, 114. https://doi.org/10.3390/v16010114.
65. Kim, J.; Seo, H.; Kim, H.W.; Kim, D.; Kwon, H.J.; Kim, Y.K. Effect of Previous COVID-19 Vaccination on Humoral Immunity 3 Months after SARS-CoV-2 Omicron Infection and Booster Effect of a Fourth COVID-19 Vaccination 2 Months after SARS-CoV-2 Omicron Infection. *Viruses* **2022**, *14*, 2458. https://doi.org/10.3390/v14112458.
66. Golec, M.; Fronczek, M.; Zembala-John, J.; Chrapiec, M.; Konka, A.; Wystyrk, K.; Botor, H.; Brzoza, Z.; Kasperczyk, S.; Buldak, R.J. Early and Longitudinal Humoral Response to the SARS-CoV-2 mRNA BNT162b2 Vaccine in Healthcare Workers: Significance of BMI, Adipose Tissue and Muscle Mass on Long-Lasting Post-Vaccinal Immunity. *Viruses* **2022**, *14*, 868. https://doi.org/10.3390/v14050868.
67. de Souza, W.M.; Muraro, S.P.; Souza, G.F.; Amorim, M.R.; Sesti-Costa, R.; Mofatto, L.S.; Forato, J.; Barbosa, P.P.; Toledo-Teixeira, D.A.; Bispo-Dos-Santos, K.; et al. Clusters of SARS-CoV-2 Lineage B.1.1.7 Infection after Vaccination with Adenovirus-Vectored and Inactivated Vaccines. *Viruses* **2021**, *13*, 2127. https://doi.org/10.3390/v13112127.
68. Thomopoulos, T.P.; Rosati, M.; Terpos, E.; Stellas, D.; Hu, X.; Karaliota, S.; Bouchla, A.; Katagas, I.; Antoniadou, A.; Mentis, A.; et al. Kinetics of Nucleocapsid, Spike and Neutralizing Antibodies, and Viral Load in Patients with Severe COVID-19 Treated with Convalescent Plasma. *Viruses* **2021**, *13*, 1844. https://doi.org/10.3390/v13091844.
69. Trinite, B.; Pradenas, E.; Marfil, S.; Rovirosa, C.; Urrea, V.; Tarres-Freixas, F.; Ortiz, R.; Rodon, J.; Vergara-Alert, J.; Segales, J.; et al. Previous SARS-CoV-2 Infection Increases B.1.1.7 Cross-Neutralization by Vaccinated Individuals. *Viruses* **2021**, *13*, 1135. https://doi.org/10.3390/v13061135.
70. Andreu, S.; Ripa, I.; Bello-Morales, R.; Lopez-Guerrero, J.A. Liposomal Lactoferrin Exerts Antiviral Activity against HCoV-229E and SARS-CoV-2 Pseudoviruses In Vitro. *Viruses* **2023**, *15*, 972. https://doi.org/10.3390/v15040972.
71. Poznanski, P.; Augustyniak-Bartosik, H.; Magiera-Zak, A.; Skalec, K.; Jakuszko, K.; Mazanowska, O.; Janczak, D.; Krajewska, M.; Kaminska, D. Molnupiravir When Used Alone Seems to Be Safe and Effective as Outpatient COVID-19 Therapy for Hemodialyzed Patients and Kidney Transplant Recipients. *Viruses* **2022**, *14*, 2224. https://doi.org/10.3390/v14102224.
72. Gallardo-Toledo, E.; Neary, M.; Sharp, J.; Herriott, J.; Kijak, E.; Bramwell, C.; Curley, P.; Arshad, U.; Pertinez, H.; Rajoli, R.K.R.; et al. Chemoprophylactic Assessment of Combined Intranasal SARS-CoV-2 Polymerase and Exonuclease Inhibition in Syrian Golden Hamsters. *Viruses* **2023**, *15*, 2161. https://doi.org/10.3390/v15112161.
73. Gammeltoft, K.A.; Zhou, Y.; Ryberg, L.A.; Pham, L.V.; Binderup, A.; Hernandez, C.R.D.; Offersgaard, A.; Fahnoe, U.; Peters, G.H.J.; Ramirez, S.; et al. Substitutions in SARS-CoV-2 Mpro Selected by Protease Inhibitor Boceprevir Confer Resistance to Nirmatrelvir. *Viruses* **2023**, *15*, 1970. https://doi.org/10.3390/v15091970.
74. Meng, J.R.; Liu, J.; Fu, L.; Shu, T.; Yang, L.; Zhang, X.; Jiang, Z.H.; Bai, L.P. Anti-Entry Activity of Natural Flavonoids against SARS-CoV-2 by Targeting Spike RBD. *Viruses* **2023**, *15*, 160. https://doi.org/10.3390/v15010160.
75. Liu, J.; Yuan, S.; Yao, Y.; Wang, J.; Scalabrino, G.; Jiang, S.; Sheridan, H. Network Pharmacology and Molecular Docking Elucidate the Underlying Pharmacological Mechanisms of the Herb Houttuynia cordata in Treating Pneumonia Caused by SARS-CoV-2. *Viruses* **2022**, *14*, 1588. https://doi.org/10.3390/v14071588.
76. Panatto, D.; Orsi, A.; Bruzzone, B.; Ricucci, V.; Fedele, G.; Reiner, G.; Giarratana, N.; Domnich, A.; Icardi, G.; Stx Study, G. Efficacy of the Sentinox Spray in Reducing Viral Load in Mild COVID-19 and Its Virucidal Activity against Other Respiratory Viruses: Results of a Randomized Controlled Trial and an In Vitro Study. *Viruses* **2022**, *14*, 1033. https://doi.org/10.3390/v14051033.
77. Taylor, R.; Bowen, R.; Demarest, J.F.; DeSpirito, M.; Hartwig, A.; Bielefeldt-Ohmann, H.; Walling, D.M.; Mathis, A.; Babu, Y.S. Activity of Galidesivir in a Hamster Model of SARS-CoV-2. *Viruses* **2021**, *14*, 8. https://doi.org/10.3390/v14010008.
78. D’Accolti, M.; Soffritti, I.; Bonfante, F.; Ricciardi, W.; Mazzacane, S.; Caselli, E. Potential of an Eco-Sustainable Probiotic-Cleaning Formulation in Reducing Infectivity of Enveloped Viruses. *Viruses* **2021**, *13*, 2227. https://doi.org/10.3390/v13112227.
79. Diomede, L.; Baroni, S.; De Luigi, A.; Piotti, A.; Lucchetti, J.; Fracasso, C.; Russo, L.; Bonaldo, V.; Panini, N.; Filippini, F.; et al. Doxycycline Inhibition of a Pseudotyped Virus Transduction Does Not Translate to Inhibition of SARS-CoV-2 Infectivity. *Viruses* **2021**, *13*, 1745. https://doi.org/10.3390/v13091745.
80. Matsuura, R.; Lo, C.W.; Wada, S.; Somei, J.; Ochiai, H.; Murakami, T.; Saito, N.; Ogawa, T.; Shinjo, A.; Benno, Y.; et al. SARS-CoV-2 Disinfection of Air and Surface Contamination by TiO_2_ Photocatalyst-Mediated Damage to Viral Morphology, RNA, and Protein. *Viruses* **2021**, *13*, 942. https://doi.org/10.3390/v13050942.
81. Urda, L.; Kreuter, M.H.; Drewe, J.; Boonen, G.; Butterweck, V.; Klimkait, T. The Petasites hybridus CO(2) Extract (Ze 339) Blocks SARS-CoV-2 Replication In Vitro. *Viruses* **2022**, *14*, 106. https://doi.org/10.3390/v14010106.
82. Carrouel, F.; Valette, M.; Perrier, H.; Bouscambert-Duchamp, M.; Dussart, C.; Tramini, P.; Bourgeois, D. Performance of Self-Collected Saliva Testing Compared with Nasopharyngeal Swab Testing for the Detection of SARS-CoV-2. *Viruses* **2021**, *13*, 895. https://doi.org/10.3390/v13050895.
83. Wagner, G.E.; Totaro, M.G.; Volland, A.; Lipp, M.; Saiger, S.; Lichtenegger, S.; Forstner, P.; von Laer, D.; Oberdorfer, G.; Steinmetz, I. A Novel High-Throughput Nanopore-Sequencing-Based Strategy for Rapid and Automated S-Protein Typing of SARS-CoV-2 Variants. *Viruses* **2021**, *13*, 2548. https://doi.org/10.3390/v13122548.
84. von Possel, R.; Menge, B.; Deschermeier, C.; Fritzsche, C.; Hemmer, C.; Geerdes-Fenge, H.; Loebermann, M.; Schulz, A.; Lattwein, E.; Steinhagen, K.; et al. Performance Analysis of Serodiagnostic Tests to Characterize the Incline and Decline of the Individual Humoral Immune Response in COVID-19 Patients: Impact on Diagnostic Management. *Viruses* **2024**, *16*, 91. https://doi.org/10.3390/v16010091.
85. Midorikawa, R.; Nakama, M.; Furukawa, H.; Oka, S.; Higuchi, T.; Nagai, H.; Nagai, N.; Tohma, S. Detection of SARS-CoV-2 Nucleocapsid, Spike, and Neutralizing Antibodies in Vaccinated Japanese. *Viruses* **2022**, *14*, 965. https://doi.org/10.3390/v14050965.
86. Kittel, M.; Eichner, R.; Aida, S.; Bode, A.; Ast, V.; Kessler, A.; Neumaier, M.; Wolfel, R.; Haselmann, V. Results of a European-Wide External Quality Assessment (EQA) Scheme for Serological Detection of Anti-SARS-CoV-2 (CoVimm)-Pitfalls of Routine Application. *Viruses* **2022**, *14*, 1662. https://doi.org/10.3390/v14081662.
87. Antonanzas, J.M.; Perramon, A.; Lopez, C.; Boneta, M.; Aguilera, C.; Capdevila, R.; Gatell, A.; Serrano, P.; Poblet, M.; Canadell, D.; et al. Symptom-Based Predictive Model of COVID-19 Disease in Children. *Viruses* **2021**, *14*, 63. https://doi.org/10.3390/v14010063.
88. Mair, T.; Ivankovic, M.; Paar, C.; Salzer, H.J.F.; Heissl, A.; Lamprecht, B.; Schreier-Lechner, E.; Tiemann-Boege, I. Processing Hundreds of SARS-CoV-2 Samples with an In-House PCR-Based Method without Robotics. *Viruses* **2021**, *13*, 1712. https://doi.org/10.3390/v13091712.
89. Brandolini, M.; Taddei, F.; Marino, M.M.; Grumiro, L.; Scalcione, A.; Turba, M.E.; Gentilini, F.; Fantini, M.; Zannoli, S.; Dirani, G.; et al. Correlating qRT-PCR, dPCR and Viral Titration for the Identification and Quantification of SARS-CoV-2: A New Approach for Infection Management. *Viruses* **2021**, *13*, 1022. https://doi.org/10.3390/v13061022.
90. Cruceriu, D.; Baldasici, O.; Balacescu, L.; Gligor-Popa, S.; Flonta, M.; Man, M.A.; Visan, S.; Vlad, C.; Trifa, A.P.; Balacescu, O.; et al. Critical Aspects Concerning the Development of a Pooling Approach for SARS-CoV-2 Diagnosis Using Large-Scale PCR Testing. *Viruses* **2021**, *13*, 902. https://doi.org/10.3390/v13050902.
91. Hitzenbichler, F.; Bauernfeind, S.; Salzberger, B.; Schmidt, B.; Wenzel, J.J. Comparison of Throat Washings, Nasopharyngeal Swabs and Oropharyngeal Swabs for Detection of SARS-CoV-2. *Viruses* **2021**, *13*, 653. https://doi.org/10.3390/v13040653.
92. Borges, V.; Isidro, J.; Macedo, F.; Neves, J.; Silva, L.; Paiva, M.; Barata, J.; Catarino, J.; Ciobanu, L.; Duarte, S.; et al. Nosocomial Outbreak of SARS-CoV-2 in a “Non-COVID-19” Hospital Ward: Virus Genome Sequencing as a Key Tool to Understand Cryptic Transmission. *Viruses* **2021**, *13*, 604. https://doi.org/10.3390/v13040604.
93. Moreira, A.C.; Teles, M.J.; Silva, T.; Bento, C.M.; Alves, I.S.; Pereira, L.; Guimaraes, J.T.; Porto, G.; Oliveira, P.; Gomes, M.S. Iron Related Biomarkers Predict Disease Severity in a Cohort of Portuguese Adult Patients during COVID-19 Acute Infection. *Viruses* **2021**, *13*, 2482. https://doi.org/10.3390/v13122482.
94. Fonseca, W.; Asai, N.; Yagi, K.; Malinczak, C.A.; Savickas, G.; Johnson, C.C.; Murray, S.; Zoratti, E.M.; Lukacs, N.W.; Li, J.; et al. COVID-19 Modulates Inflammatory and Renal Markers That May Predict Hospital Outcomes among African American Males. *Viruses* **2021**, *13*, 2415. https://doi.org/10.3390/v13122415.
95. Erman, A.; Wechtersbach, K.; Velkavrh, D.; Plesko, J.; Frelih, M.; Kojc, N. Just Seeing Is Not Enough for Believing: Immunolabelling as Indisputable Proof of SARS-CoV-2 Virions in Infected Tissue. *Viruses* **2021**, *13*, 1816. https://doi.org/10.3390/v13091816.
96. Bennett, R.S.; Postnikova, E.N.; Liang, J.; Gross, R.; Mazur, S.; Dixit, S.; Kocher, G.; Yu, S.; Georgia-Clark, S.; Gerhardt, D.; et al. Scalable, Micro-Neutralization Assay for Assessment of SARS-CoV-2 (COVID-19) Virus-Neutralizing Antibodies in Human Clinical Samples. *Viruses* **2021**, *13*, 893. https://doi.org/10.3390/v13050893.
97. Doenhardt, M.; Gano, C.; Sorg, A.L.; Diffloth, N.; Tenenbaum, T.; von Kries, R.; Berner, R.; Armann, J.P. Burden of Pediatric SARS-CoV-2 Hospitalizations during the Omicron Wave in Germany. *Viruses* **2022**, *14*, 2102. https://doi.org/10.3390/v14102102.
98. Morgan, G.; Casalino, S.; Chowdhary, S.; Frangione, E.; Fung, C.Y.J.; Haller, S.; Lapadula, E.; Scott, M.; Wolday, D.; Young, J.; et al. Characterizing Risk Factors for Hospitalization and Clinical Characteristics in a Cohort of COVID-19 Patients Enrolled in the GENCOV Study. *Viruses* **2023**, *15*, 1764. https://doi.org/10.3390/v15081764.
99. Flisiak, R.; Rzymski, P.; Zarebska-Michaluk, D.; Ciechanowski, P.; Dobrowolska, K.; Rogalska, M.; Jaroszewicz, J.; Szymanek-Pasternak, A.; Rorat, M.; Kozielewicz, D.; et al. Variability in the Clinical Course of COVID-19 in a Retrospective Analysis of a Large Real-World Database. *Viruses* **2023**, *15*, 149. https://doi.org/10.3390/v15010149.
100. Kala, M.; Ahmad, S.; Dhebane, M.; Das, K.; Raturi, M.; Tyagi, M.; Kusum, A. A Cross-Sectional Comparative Characterization of Hematological Changes in Patients with COVID-19 Infection, Non-COVID Influenza-like Illnesses and Healthy Controls. *Viruses* **2022**, *15*, 134. https://doi.org/10.3390/v15010134.
101. Muller, M.; Volzke, J.; Subin, B.; Schmidt, C.J.; Geerdes-Fenge, H.; Reisinger, E.C.; Muller-Hilke, B. Distinguishing Incubation and Acute Disease Stages of Mild-to-Moderate COVID-19. *Viruses* **2022**, *14*, 203.https://doi.org/10.3390/v14020203.

